# Discrimination of Tryptophan Enantiomers at Sub‐pm Level by Multiparametric Analysis of a Label‐Free Organic Immunosensor

**DOI:** 10.1002/smtd.202500545

**Published:** 2025-08-15

**Authors:** Matteo Genitoni, Pierpaolo Greco, Alessandro Paradisi, Matteo Sensi, Marcello Berto, Mauro Murgia, Michele Di Lauro, Carlo Augusto Bortolotti, Luciano Fadiga, Fabio Biscarini

**Affiliations:** ^1^ Center for Translational Neurophysiology of Speech and Communication Istituto Italiano di Tecnologia Ferrara 44121 Italy; ^2^ Department of Neuroscience and Rehabilitation Università di Ferrara Ferrara 44121 Italy; ^3^ Life Science Department Università di Modena e Reggio Emilia Modena 41125 Italy; ^4^ Organic Bioelectronics Srl Ferrara 44121 Italy; ^5^ Institute for the Study of Nanostructured Materials (ISMN) Consiglio Nazionale delle Ricerche Bologna 40129 Italy

**Keywords:** electrode functionalization, enantioselection, organic electronics, principal component analysis, tryptophan

## Abstract

Electrolyte‐gated organic transistors (EGOTs) exponentially amplify minute polarization changes at the gate electrode into the channel current. Antibodies grafted on the EGOT gate electrode enable specific recognition of target species, yet this strategy may not be sufficient per se to resolve the target from its antagonists. Here, a label‐free EGOT immunosensor is functionalized with the antibody anti‐L‐enantiomer of Tryptophan (Trp), exhibiting sensitivity to Trp chirality. Nevertheless, the relative current change in transfer curves does not unambiguously differentiate L from D enantiomers in the concentration range 1 fm to 10 nm. To overcome this limitation, a multivariate principal component analysis (PCA) is applied on the set of renormalized parameters, extracted from the whole transfer curves according to our recent EGOT model: both L and D enantiomers are neatly separated by the sign of their principal components. Enantiomeric discrimination onset is 1 and 10 pm at 90% level of confidence and prediction, respectively, at least one order of magnitude lower than the enantiodiscrimination levels previously reported with EGOT biosensor. The same analysis performed on the best fit dose curves allows to discriminate L and D enantiomers down to the unprecedented level of detection of 100 fm.

## Graphical abstract

Multiparametric analysis is a crucial aspect for enhancing the final sensitivity of biosensors based on organic transistors. The traditional approaches to treat the experimental data are not always suitable to extract the correct response. In this work EGOT characteristics are processed to achieve enantiodiscrimination of free Tryptophan enantiomers down to the unprecedented level of detection of 100 fm.

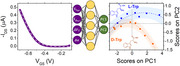

## 1 Introduction

Chirality is a fundamental molecular property in biorecognition.^[^ ^1^ ^]^ As the origin of homochirality remains unclear, biological systems exhibit remarkable selectivity for chiral molecules, such as peptides, carbohydrates, and nucleic acids, to regulate cellular metabolic processes.^[^ ^2^ ^]^ Enantiomers exhibit different pharmacological, pharmacokinetic, and toxicological activities,^[^ ^3^ ^]^ and is important to resolve the chirality of synthetic molecules interacting with living systems prior to their administration.

Amino acids, except glycine that is achiral, are among the smallest chiral molecules in Nature.^[^ ^4^ ^]^ The L‐amino acids are the building blocks in proteins synthesis, while D‐amino acids can be incorporated into proteins only via modified ribosomes.^[^ ^5^ ^]^ Among the L‐amino acids, L‐Tryptophan (L‐Trp) plays a crucial role in the L‐Kynurenine pathway, and in the Serotonin pathway.^[^ ^6^ ^]^ Alterations in the physiological concentration of L‐Tryptophan (L‐Trp) (4 µm in blood, 2 µm in cerebrospinal fluid)^[^ ^7^ ^]^ were correlated with pathological disorders, including cancer,^[^ ^8^ ^]^ depression,^[^ ^9^ ^]^ Alzheimer's disease,^[^ ^10^ ^]^ and AIDS‐related dementia complex.^[^ ^11^ ^]^

The other enantiomer, D‐Tryptophan (D‐Trp), albeit present in microorganisms, does not participate in metabolic pathways in the human body^[^ ^12^ ^]^; however, D‐Trp plays a significant role as an intermediate in the synthesis of peptide antibiotics and as an immunosuppressant in the pharmaceutical industry.^[^ ^13^ ^]^

Trp derivatives (e.g. serotonin, melatonin, and halogenated Trp) play a crucial role in the food, chemical (polymers, pesticides) and pharmaceutical industries.^[^ ^14^ ^]^ Since the biosynthesis of these compounds originates from enantiomerically pure Trp, the discrimination between enantiomers is of paramount importance to enhance both the efficacy and safety of the production processes. Currently, a variety of analytical methods, including high‐performance liquid chromatography (HPLC),^[^ ^15^, ^16^, ^17^ ^]^ colorimetry,^[^ ^18^ ^]^ capillary electrophoresis combined with circular dichroism,^[^ ^19^ ^]^ and electrochemical analysis^[^ ^20^, ^21^, ^22^ ^]^ are employed for enantiomeric discrimination. A detailed comparison of the techniques successfully applied for chiral recognition of Trp enantiomers is reported in Table S1 (Supporting Information).^[^ ^15^, ^16^, ^17^, ^18^, ^19^, ^20^, ^21^, ^22^, ^23^, ^24^, ^25^, ^26^, ^27^, ^28^, ^29^, ^30^, ^31^, ^32^, ^33^, ^34^, ^35^, ^36^, ^37^, ^38^, ^39^, ^40^ ^]^ Notably, only a few studies^[^ ^23^, ^24^, ^25^, ^26^, ^27^ ^]^ have explored Trp concentrations below physiological levels. Enantiomeric discrimination in the nanomolar range was achieved with high‐cost, time‐consuming techniques such as UV spectroscopy and surface plasmon resonance (SPR), which require sophisticated laboratory equipment and highly trained personnel.^[^ ^23^, ^24^, ^25^ ^]^ The remaining two studies that explore low‐concentration ranges are based on more portable and user‐friendly transistor platforms.^[^ ^26^, ^27^ ^]^ Specifically, Li and co‐workers developed a MOSFET capable of partial enantiodiscrimination in the nanomolar range, while Zhang et al. implemented a molecularly imprinted polymer (MIP) on the gate electrode of an electrolyte gated organic transistor (EGOT), achieving chiral discrimination at a concentration of a few µm. To the best of our knowledge, there were no studies of chiral discrimination at concentrations smaller than nm.

EGOTs were demonstrated as robust ultrasensitive label‐free biosensors.^[^ ^41^, ^42^, ^43^ ^]^ A variety of clinically relevant molecular targets^[^ ^44^ ^]^ detected with EGOT include neurotransmitters,^[^ ^45^ ^]^ metabolites,^[^ ^46^ ^]^ nucleic acid,^[^ ^47^, ^48^ ^]^ inflammatory cytokines,^[^ ^49^ ^]^ proteins biomarkers,^[^ ^50^, ^51^, ^52^ ^]^ antidrug antibodies,^[^ ^53^ ^]^ viruses,^[^ ^54^, ^55^ ^]^ bacteria.^[^ ^56^ ^]^ The common thread of these investigations is that the sizes of probe and target are comparable, while the length scale from the surface where recognition occurs scales as the size of the probe. Examples are aptamers grafted on the electrode surface that recognize inflammatory proteins, like cytokines.^[^ ^49^ ^]^ Recognition of large targets with small probes was also reported, while the opposite is rarer.

Chiral discrimination of small molecules with EGOTs was reported in the literature a few times. Torsi et al.^[^ ^57^ ^]^ use a chiral semiconductor thin film cast on top of the channel of an OFET to resolve the enantiomers of carvone and citronellol. Mulla and co‐workers reported an EGOT with a gate electrode functionalized with porcine odorant binding protein, which demonstrated the ability to distinguish between (S)‐ and (R)‐carvone enantiomers at concentrations higher than 100 pm.^[^ ^58^ ^]^

In this work, we investigate the recognition of free Trp in solution with EGOT immunosensor functionalized with the anti‐L‐Trp antibody thus in conditions of weak coupling between the biorecognition and the electrode. We explore Trp recognition at the 1 fm–10 nm concentration range, which is much lower than the physiological concentration of L‐Trp.^[^ ^7^ ^]^

We compare two antibody anchoring strategies for the anti‐L‐Trp electrode: the Schiff base reaction (SBR) and EDC‐NHS activation of the amide bond. Interestingly, we demonstrate that the SBR functionalization protocol is leading to increased sensitivity for L‐Trp, compared to EDC‐NHS. Furthermore, while the EDC‐NHS approach yields a response solely in terms of current and transconductance variation, the SBR functionalization enables a multiparametric response upon Trp exposure.

In the second part of the study, we leverage the high sensitivity and the multiparametric analysis of parameters extracted from EGOT response, to address the discrimination between the L‐ and D‐enantiomers of Trp. Although the average values exhibit monotonic trends with concentration, standard univariate analysis of the transfer curves does not reveal statistically significant differences between the two enantiomeric forms.

To overcome this limitation, we propose a novel and straightforward approach to analyze EGOT biosensor data by combining the multiparametric response extracted from the transfer curves with multivariate analysis based on principal component analysis (PCA). Discrimination between L‐ and D‐Trp is achieved at concentrations above 10 pm using PCA of the experimental data. Remarkably, when PCA is applied to the dose–response curves, the discrimination threshold shifts down to the sub‐pm range (10–100 fm), several orders of magnitude lower than previously reported.

## 2 Results and Discussion

The EGOT device for the biosensing experiment is based on an extended gate architecture previously reported by our group. The interdigitated source and drain electrodes and the gate electrode are fabricated on separate substrates, and the planar gate is a few cm distance from the channel, connected to the semiconducting channel by means of thin tubing containing electrolyte.^[^ ^47^, ^59^ ^]^

**Figure 1:**
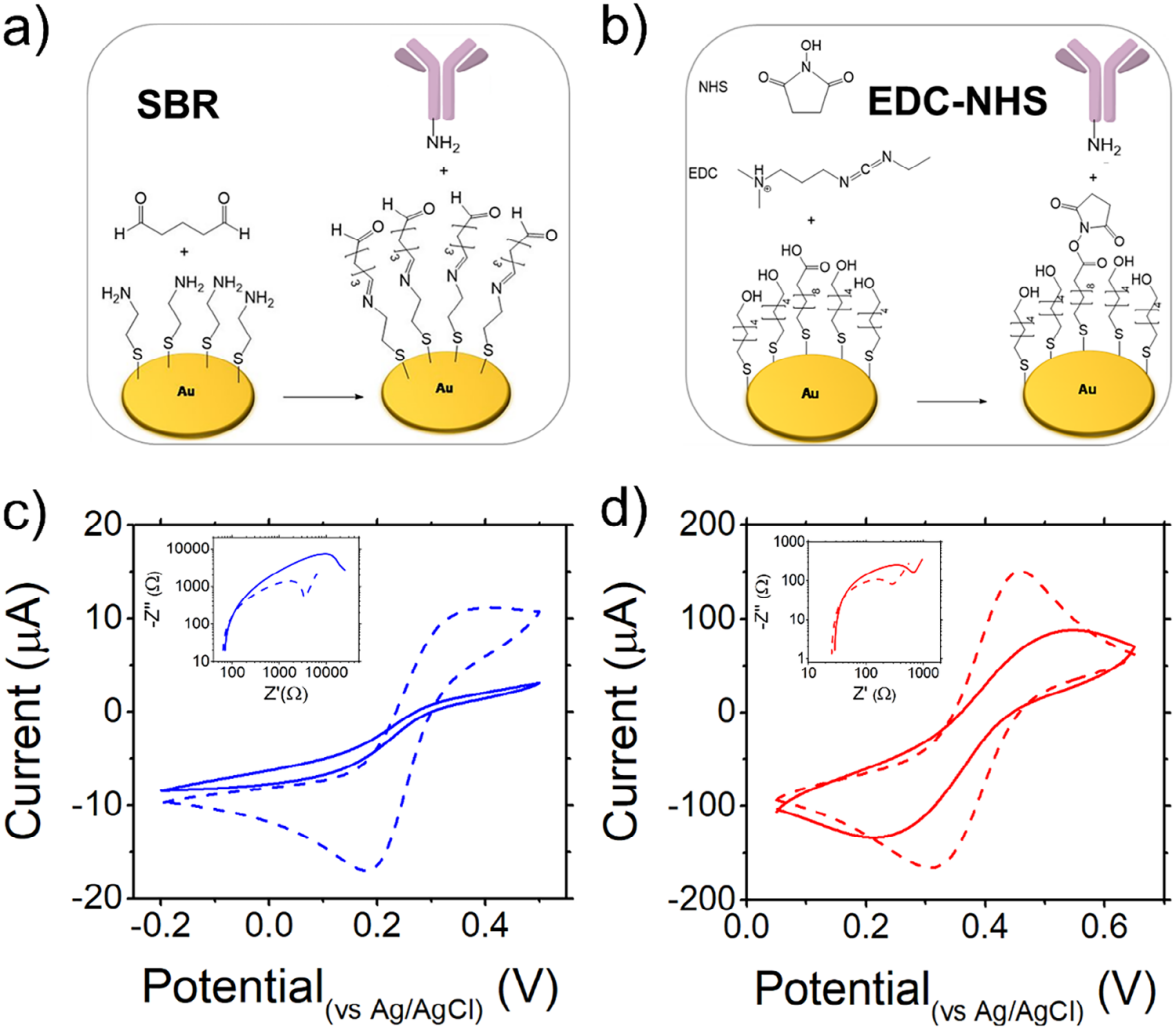
a) SBR functionalization shows the gold substrate coated with cysteamine, followed by the formation of the imide (Schiff base) with glutaraldehyde GA. b) EDC‐NHS functionalization shows the formation of a reactive layer stack before antibody anchoring. c) Electrochemical characterization of SBR and d) EDC/NHS functionalization: cyclic voltammograms before (dashed line) and after (continuous line) incubation with anti‐L‐Trp antibody; inset, impedance spectroscopy Nyquist's plot (double log plot) evidences the increase of the impedance upon antibody binding.

The gate electrode functionalization was carried out according to two functionalization processes, schematically depicted in **Figure** **1**. Details can be found in refs. [60, 61]. The main reaction steps of Schiff base functionalization^[^ ^60^, ^62^ ^]^ leads to an imide bond formed between the aldehydic group of glutaraldehyde (GA) and the amino group of L‐Trp antibody. The N‐(3‐dimemethylaminopropyl)‐N‐ethylcarbodiimide (EDC)‐N‐Hydroxysuccinimide (NHS) reaction activates carboxylic group of the 11‐mercaptoundecanoic acid self‐assembled monolayer (SAM) to form the amide bond with the anti‐L‐Trp antibody terminal amino group.^[^ ^61^, ^63^ ^]^ After the anchoring of the antibody, the unreacted groups in the SAMs of either SBR or EDC‐NHS are blocked with ethanolamine.

The functionalization of the gold electrodes is assessed by cyclic voltammetry (CV) and electrochemical impedance spectroscopy (EIS) measurements, in presence of a K_3_[Fe(CN)_6_] redox probe (see Experimental section for other details). The voltammograms shown in Figure 1c,d indicate that the current values displayed for SBR are one order of magnitude lower than EDC‐NHS since the GA layer may be more effectively covering the gold electrodes. The insets of Figure 1c,d show the Nyquist plots of the respective impedance measurements. In the inset, the magnified curves highlight the characteristic semicircle profile of the resistive interface. The imaginary part of impedance increases almost 5 times reaching ≈7.5 kΩ for SBR, and 2.5 times for EDC‐NHS. This confirms that the functionalization yields compact functionalization layers.

**Figure 2:**
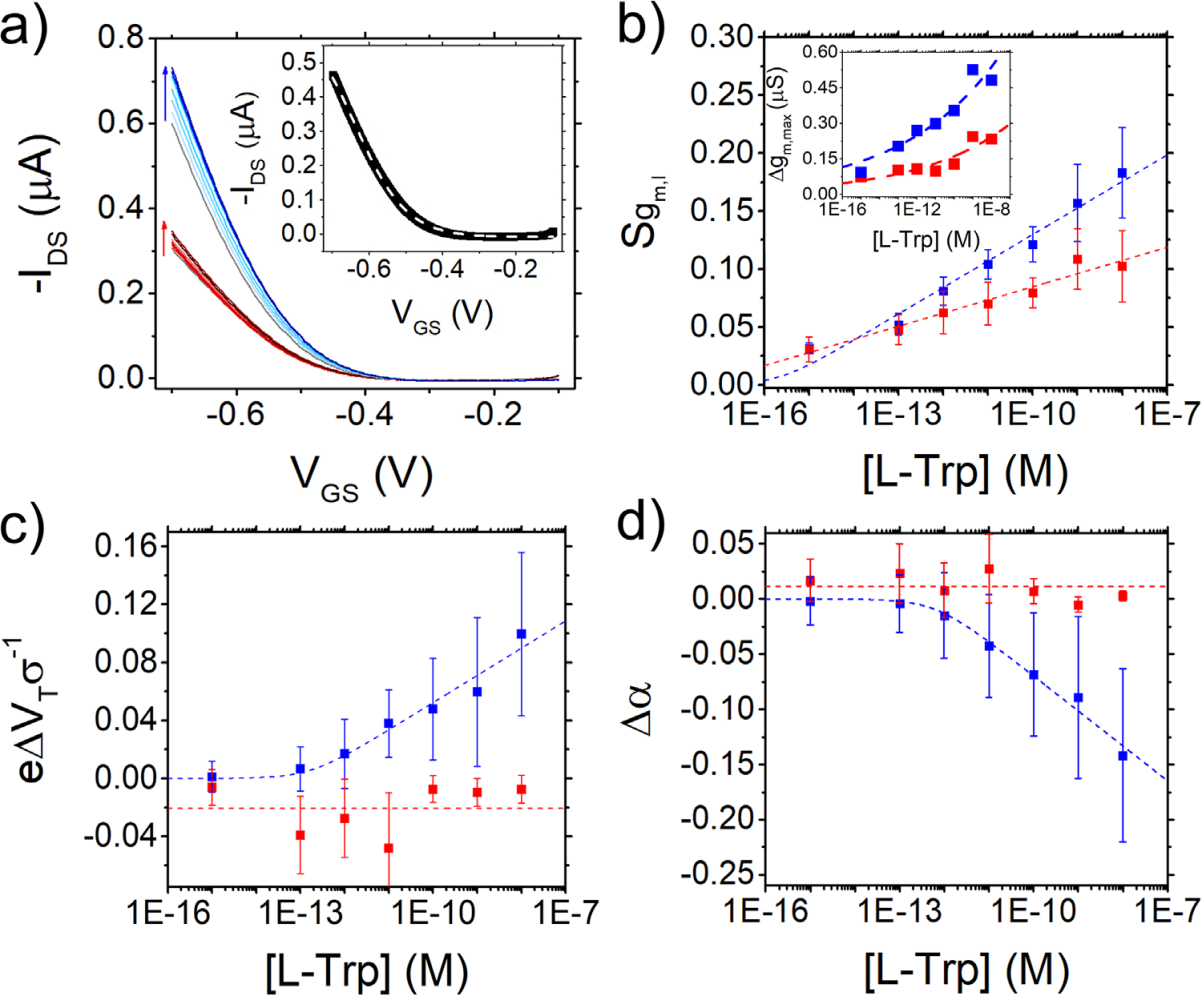
a) Representative transfer curves of EGOT device (V_DS_ = −0.2 V) with gate electrode functionalized with SBR (blue) and with the EDC‐NHS protocol (red) upon interaction with L‐Trp. The inset shows the variation of the average maximum value of the transconductance. b) Semilog plot of the signal $S_{g_{\mathrm{ml}}}$ vs [L‐Trp]. c) Semilog plot of the turn‐on voltage shift ΔV_T_ vs [L‐Trp]. d) Semilog plot of the shift of the parameter α vs [L‐Trp]. Dashed lines are guide‐to‐the‐eye obtained by best fit with ULM Equation 3. In the inset of Figure 2a a representative transfer curve is fitted according to Equation 1. In the inset of Figure 2b the dashed line is power law guide to the eye to describe Δg_m,max_ vs [Trp].

**Figure** **2**a displays the response of EGOT biosensor for SBR (blue curves) and EDC‐NHS (red curves) strategy to the increasing concentration [L‐Trp]. The current increase for both functionalization strategies could be explained by considering that L‐Trp molecules are negatively charged (*pI_L‐Trp_ = 5.89*)^[^ ^64^ ^]^ at the experimental pH = 7.4. Their binding to the functionalized electrode makes the effective gate voltage more negative and hence increases the carrier density in the p‐type channel.^[^ ^50^, ^65^ ^]^ The magnitude of the *I_DS_* shift cannot be attributed solely to the charge of the target analyte as it is clearly dependent on the functionalization strategy adopted. The functionalization procedure affects the density and binding distance of the molecular target to the antibody. For the SBR functionalized electrodes, the transfer curves shift up by ≈20% for L‐Trp concentration increasing from 1 fm up to 10 nm. Comparatively, for the EDC‐NHS functionalized electrode, the current increase is 15% lower in the same range. To quantify the current variation of the EGOT biosensor when exposed to increasing concentration of the analyte, we built a calibration dose curve by calculating the recorded current signal against the target  concentration. The signal is defined as the normalized current variation with respect to the blank sample $S_{I_{DS}}\left(\left[L-Trp\right]\right)=\left\langle \frac{I_{DS}\left(\left[L-Trp\right]\right)-I_{DS}\left(0M\right)}{I_{DS}\left(0M\right)}\right\rangle \;$, where *I_DS_* ([*L* − *Trp*]) is the current recorded at a fixed gate potential and *I_DS_* (0*M*) is the current measured at the same gate potential in the blank sample.

We recently proposed a model to fit the whole experimental transfer curves^[^ ^66^ ^]^ with the following EGOT current expression:

(1) $$\begin{array}{ccc} I_{DS} & & \left(V_{GS};V_{DS}\right)\approx I_{DS,off}\left(V_{DS}\right)\\ & & +\;\;g_{m,l}\left\{\frac{\alpha \sigma }{e}\frac{sinh\left(2e\frac{V_{GS}-V_{T}}{\sigma }\right)-sinh\left(e\frac{V_{GS}-V_{T}}{\sigma }\right)}{sinh\left(\frac{\Delta E_{gap}}{2\sigma }\right)+\alpha cosh\left(2e\frac{V_{GS}-V_{T}}{\sigma }\right)}\right\} \end{array}$$

where *e* is the elementary charge, *V_T_* is the switch‐on voltage, *σ* is the energy disorder of the density of states (DOS) of the organic semiconductor channel, its bandgap Δ*E_gap_* is a material property, and the parameter $\alpha =\frac{2e^{2}dn_{max}}{C_{DL}\sigma }\;$ is the ratio between the maximum of areal charge carrier density *2edn_max_* in the DOS tail and the interfacial charge carrier density *C_DL_* σ/*e*. The energy disorder *σ* embodies the variations of morphology of the thin films whose thickness is *d*. The EGOT response is thus represented by the variation of the free parameters *g*_*m*,*l*_,  α,  *V_T_* vs [Trp]. The accuracy in fitting the nonlinear variation of *I_DS_* as a function of *V_GS_* is shown in the inset of Figure 2a, where a transfer curve is overlaid with the best fit *I_DS_* as from Equation 1.

In Figure 2b we show the signal $S_{g_{m,l}}\left(\left[L-Trp\right]\right)=\left\langle \frac{g_{m,l}\left(\left[L-Trp\right]\right)-g_{m,l}\left(0M\right)}{g_{m,l}\left(0M\right)}\;\right\rangle$, defined as the relative variation of the linear transconductance $g_{m,l}=\frac{W}{L}\;\mu C_{DL}V_{DS}$ extracted as a best fit parameter of Equation 1.^[^ ^66^ ^]^ The slope of the transfer curves far from the switch‐on voltage is higher for the SBR functionalized electrodes than the EDC‐NHS functionalized ones. The inset of Figure 2b shows instead the increasing trend of the maximum transconductance, taken as the first derivative $g_{m,max}=\;\max\left(\frac{dI_{DS}}{dV_{GS}}\right)$ of the *I_DS_* current in Figure 2a. In Figure 2c, we show the dimensionless shift of the turn‐on voltage *e*Δ*V_T_* ([*L* − *Trp*])/σ = *e*〈*V_T_* ([*L* − *Trp*]) − *V_T_* (0*M*)〉/σ and in Figure 2d the dimensionless shift of Δα ([*L* − *Trp*]) = 〈α([*L* − *Trp*]) − α(0*M*)〉. The displayed error bars are estimated as standard error of the mean (SEM), calculated according to the definition:

(2) $$SEM=\frac{1}{\sqrt{N}}\sqrt{\left(\frac{\sum_{j=1}^{N}{\left(x_{j}-\bar{X}\right)}^{2}}{N-1}\right)}$$

SEM provides a more precise and robust estimate of the deviation from the mean parameter values. For each concentration step, sample means are obtained from modelled transfer curves, computed as the average of the last three curves for each of the four datasets analyzed.^[^ ^67^ ^]^ We observe that the SEM increases with signal amplitude, a characteristic feature of exponentially varying observables, consistent with the functional form of the signal in our framework.

To describe the binding process between the Tryptophan molecule with the anti L‐Trp antibody, we model the monotonic trend of the signal $S_{g_{m,l}}$ vs the logarithm of [L‐Trp] with an isotherm function. Among several isotherms we assessed, the Uniform Langmuir Model (ULM),^[^ ^50^, ^61^, ^68^ ^]^ yields the most accurate description of the $S_{g_{m,l}}$ vs [L‐Trp] trend (R^2^ COD = 0.98 for SBR (pH = 10), R^2^ COD = 0.97 for EDC‐NHS). The ULM model accounts for a distribution of binding sites on the electrode surface, characterized by a binding energy interval *U* ∈ [*U_min_; U_max_*]. The distribution of binding sites energies may arise from the random orientation of antibodies, present in both SBR and EDC‐NHS, or from the local inhomogeneity of the thin film, leading to a heterogeneous energy landscape across the gate electrode surface. The ULM isotherm reads:

(3) $$S_{X}=\frac{S_{X,max}}{2A}ln\left\{\frac{1+K_{avg}\left[L-Trp\right]exp\left(A\right)}{1+K_{avg}\left[L-Trp\right]exp\left(-A\right)}\right\}$$

where *S_max_* is the plateau reached at [L‐Trp]>10 nm; *K_ULM_* is the average binding constant; $A=\frac{\left(U_{max}-U_{min}\right)}{2RT}\;$ is the rescaled energy variable describing the energy distribution of the adsorption sites; *R* the universal gas constant and *T* is the absolute temperature. Assuming that the average affinity between the target analyte and the antibody does not depend on the functionalization strategy, we extract a best fit value *K_avg_* =  1.4(± 1.1) 10^9^ for both functionalization strategies as a global parameter. *S_max_* describes the behaviour of *S_gm,l_* at concentrations above 10 nm nm and is larger for SBR *S_max_* = 0.30(± 0.01) than that of EDC‐NHS *S_max_* = 0.20(± 0.01). The differences between the two functionalization strategies are explained by the distribution of the binding energies: for SBR A = 15.0 (± 0.9), while for EDC‐NHS *A* = 19.1(± 1.5).

We use the same functional in Equation 3 to fit also the data in Figure 2c,d. In Figure 2c the trend of the turn‐on voltage shift Δ*V_T_* vs [L‐Trp] reveals the greater sensitivity of the SBR functionalization with respect to the EDC‐NHS functionalization, which remains nearly constant vs [L‐Trp] for this parameter. In Figure 2d, the response Δα vs [L‐Trp] evidences the change of capacitance of the gate upon binding of the Trp to the antibody. The EDC‐NHS functionalized gate exhibits a weak variation of Δα with increasing L‐Trp concentration, whereas a marked variation is observed with the SBR functionalization. The α parameter being the ratio of two areal density of carriers, is subjected to sizable changes from one gate electrode to another. The change of the gate areal capacitance *C_G_* may ultimately be related to the quantity of antibody grafted to the surface, which we estimate to be higher for the SBR functionalization with respect to EDC‐NHS, as corroborated also by impedance spectroscopy reported in the insets of Figure 1c,d.

**Figure 3:**
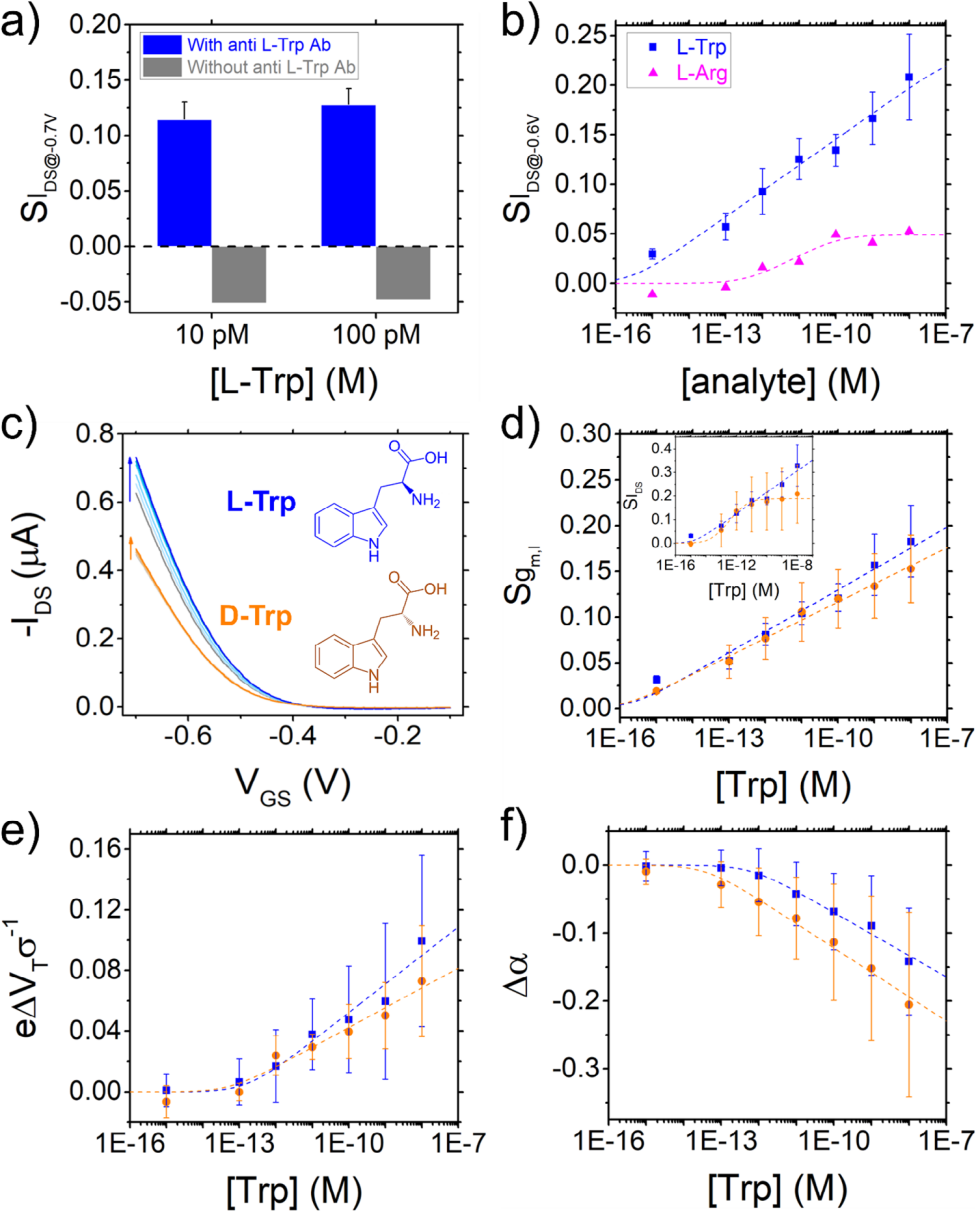
a) Signal calculated on current in control experiments performed with gate electrodes functionalized with and without the monoclonal anti‐L‐Trp antibody. b) Device response when the functionalized gate electrodes with the complete SBR protocol was exposed to L‐Arginine (magenta triangles). In the semilog plot of the signal vs [analyte], the experimental data are fitted with ULM Equation 3 as guide‐to‐the‐eye. c–f) Analysis of the response of SBR functionalized EGOT upon exposure to L‐Trp (blue markers) and D‐Trp (orange markers) solutions. c) Representative transfer curves of EGOT device (V_DS_ = −0.2 V) with the gate electrode functionalized with optimum SBR protocol. The inset shows the chemical structure of L and D Tryptophan enantiomers. d) Semilog plot of the signal $S_{g_{ml}}$ vs [Trp]. The inset shows the semilog of the current signal at *V_GS_* −0.4 V vs [Trp]. e)Semilog plot of the renormalized turn‐on voltage shift Δ*V_T_* vs [Trp]. f) Semilog plot of the shift of the parameter α vs [Trp]. Dashed lines are guide‐to‐the‐eye obtained by best fit with ULM Equation 3.

At this point, we would like to highlight that the response of the EGOT device—in terms of turn‐on voltage shift and Δα, upon exposure of the gate electrode to increasing concentrations of L‐Trp, depends on the functionalization strategy. In particular, with the SBR functionalization strategy, four distinct dose–response curves can be exploited (as from Figures 2b–d and **3**a) for the quantification of L‐Trp, whereas for EDC–NHS the only significant variations are observed in the current and transconductance signals (Figure 2b). This differing behaviour can be attributed to variations in the surface morphology of the functionalized gold electrodes prior to antibody anchoring. The morphology differences were evaluated by tapping mode atomic force microscopy (AFM) shown in Figure S1 (Supporting Information). The chemisorbed SAM activated with EDC–NHS forms a thin film with an expected thickness of a few angstroms^[^ ^69^ ^]^ and induces only a modest increase in surface roughness compared to the bare gold electrode (3.61 nm for EDC–NHS vs. 3.21 nm for bare gold). In contrast, the SBR activation protocol leads to the formation of an oligomeric film exhibiting a higher surface roughness, with an estimated value of 7.33 nm. The larger surface area of SBR apparently yields a larger number density of binding sites for grafting antibodies, leading to increased signals for SBR.

To verify whether the measured signal in the SBR functionalization strategy is indeed originated from the specific binding of L‐Tryptophan to the monoclonal antibody on the gate electrode, we conducted two control experiments: the first in the absence of antibody and the second by keeping the functionalization protocol unaltered but exposing the gate to a different amino acid, *viz*., L‐arginine. In absence of biorecognition unit, the device shows only a modest, negative response when incubated with L‐Trp (grey bars in **Figure** **3**a), with respect to positive signal reported for the specific anti L‐Trpantibody binding (blue bars). When the antibody‐functionalized gate electrode is exposed to L‐Arginine, the immunosensor provides an almost negligible response (S < 0.05) also in presence of [L‐Arg] higher than 1 nM (Figure 3b).

We now address the response of the EGOT immunosensor functionalized with the anti‐L‐Trp antibody to chirality of the two enantiomers, L‐Trp and D‐Trp. To our surprise, the antibody, which is highly selective in buffer solutions with albumin (data not shown), in the EGOT yields sizable signals for D‐Trp.

The EGOT response at different concentrations of L‐Trp and D‐Trp is displayed in Figure 3c–f. The dashed lines, serving as guide‐to‐the‐eye, are fitted according to Equation 3. In Figure 3a, we present the transfer curves *I_DS_* (Trp) vs *V_GS_* at increasing concentrations of the two enantiomeric forms, whose chemical structures are reported in the inset of Figure 3c. The curves of the D‐Trp (orange lines) are always below the ones for L‐Trp (blue), however the distance between the two bundles of curves is device‐dependent. In Figure 3d we show $S_{g_{m,l}}$(Trp) again the dose curve of L‐Trp is ≈10% higher that of D‐Trp at all concentrations, with the same ambiguity generated by the error bars overlapping the two curves. In the inset of Figure 3d it is reported the current signal $S_{I_{DS}}\left(\left[Trp\right]\right)=\frac{I_{DS}\left(\left[Trp\right]\right)-I_{DS}\left(0\;M\right)}{I_{DS}\left(0\;M\right)}\;$ estimated at *V*_GS_ = − 0.4 V. The trends of the two dose curves are comparable, with $S_{I_{DS}}$(L‐Trp) *>* $S_{I_{DS}}$(D‐Trp) although the error bars prevent the unambiguous direct assignment of a signal to one of the enantiomers below 100 pm. Dose curves are calculated also for the shifts of turn‐on voltage Δ*V_T_* (Trp) in Figure 3e and Δα(Trp) in Figure 3f. Among the parameters, Δα(Trp) appears to be the parameter allowing for better discrimination between the two chiral forms, although the error bars introduce a substantial uncertainty in the discrimination of the two enantiomers. To assess the possibility to perform enantio‐discrimination using a standard univariate analysis, we carried out a one‐way ANOVA test on each parameter reported in Figure 3 (*viz*., $S_{I_{DS}}$, $S_{g_{ml}}$, $\frac{e\Delta V_{T}}{\sigma }$, Δα) for each concentration tested. This individual analysis yielded *p*‐values greater than 0.05 for each parameter at all concentrations, thus supporting the statement that univariate analysis of the parameters does not enable a significant differentiation between the response signals of L‐Trp and D‐Trp within the concentration range explored (from 1 fm to 10 nm).

This limitation may be attributed to the partial affinity of D‐Trp towards the anti‐L‐Trp antibody. By assuming that the surface binding obey to thermodynamic equilibrium, parameters extracted from EGOT transfer curves have been demonstrated to provide a good description of antigen‐antibody affinity constant.^[^ ^61^, ^70^ ^]^ In Table S2 (Supporting Information) we report the estimated value of the affinity constant (*K_avg_*), extracted from the ULM fit for the two enantiomeric forms, for each parameter reported in Figure 3.

The *K_avg_* value extracted from $S_{g_{m,l}}$ for the binding of L‐Trp with the antibody is consistent with the affinity binding constant (*K_A_ (L‐Trp) =* (3.9 ± 0.6)E8) calculated from the ELISA test performed using the same mouse monoclonal anti L‐Trp antibody employed in the EGOT immunosensor (Figure S2a, Supporting Information). Conversely, the *K_A_* extracted from the ELISA test for D‐Trp (4.1 ± 2.1)E6 is lower than the *K_avg_* estimated from $S_{g_{m,l}}$ dose curve for D‐Trp (Table S2, Supporting Information). Such a difference may be explained by the derivatization process involving other proteins, which was applied to the Trp enantiomers prior to the competitive ELISA assay (see Experimental section for details). Furthermore, the *K_A_ (D‐Trp)* is lower than *K_A_ (L‐Trp)* but clearly not zero, indicating a weaker, yet still detectable, affinity of D‐Trp for the anti‐L‐Trp antibody. This partial (non‐zero) affinity of D‐Trp was also confirmed using an ELISA kit developed to quantify Trp in real matrices and using a polyclonal rabbit anti‐L‐Trp antibody, as shown in Figure S2b (Supporting Information).

**Figure 4:**
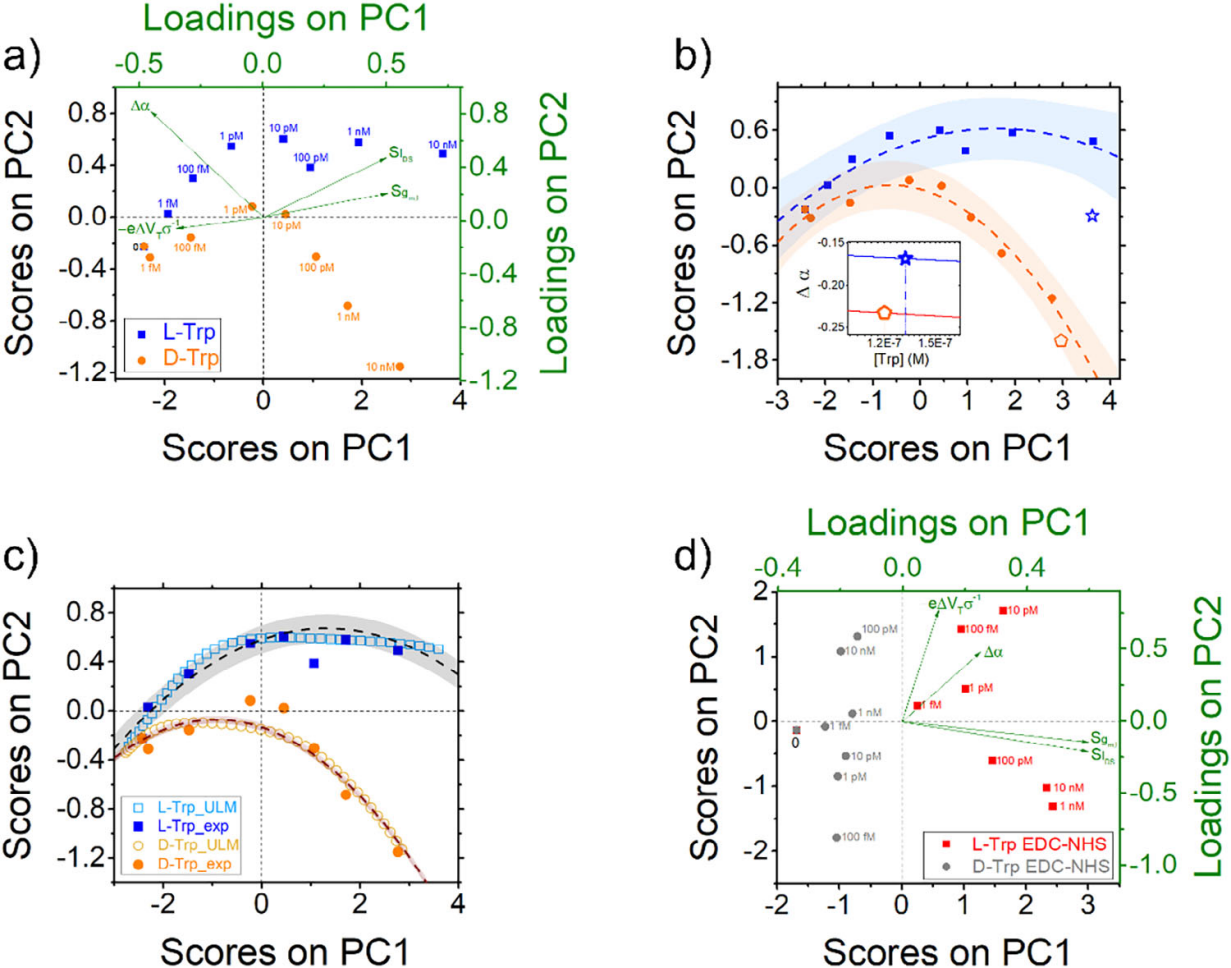
PCA analysis of the data from the L‐Trp EGOT sensor. a) Scores (black axes) and loadings (green axes) biplot obtained from the PCA analysis from SBR functionalized EGOTs. The data relative to the two enantiomers are blue squares (L) and orange circles (D) markers, respectively, with the corresponding concentrations indicated. The green arrows indicate the vector directions of the loadings of the four dimensionless parameters extracted. b) Same data with their respective parabolic fit (continuous lines) and their prediction ranges at 90% level (shadowed areas). Two unknown concentration samples (star and pentagon) assigned to L or D clusters in the PC biplot and later correlated to the points on the dose curve Δα vs [Trp] (inset) yielding their concentration. c) PCA score plot computed obtained by combining all points of the best fit curves of the four variables shown in Figure 3 (empty markers, ULM for the dose curves) superimposed to the experimental ones (filled markers, exp). Dashed line and shaded area represent parabolic fit and prediction at 90% respectively. d) PCA score plot as in (a) extracted from the experiments with EDC/NHS functionalized gate electrodes.

To identify an optimum criterion that would allow us to unambiguously discriminate L‐Trp from D‐Trp at low concentrations, we combine all the renormalized parameters extracted from transfer curves into Principal Component Analysis (PCA), including the data sets for both L‐Trp (blue) and D‐Trp (orange) (**Figure** **4**). PCA, a bilinear decomposition/projection technique able to condense large amounts of data into few parameters, was successfully exploited for multivariate analysis in biosensing.^[^ ^50^, ^71^, ^72^, ^73^, ^74^ ^]^ Further details are reported in Experimental section.

In Figure 4a, we report the biplot (*viz*., score and loading) of PCA obtained from SBR data set by using as input variables the three renormalized model parameters, displayed in Figure 3b–d, and the current signal $S_{I_{DS}}\;$ at *V*_GS_ = −0.4 V (in the subthreshold regime). The score plot, black axes, of PC1 and PC2 (which accounts for 98.5% of variance) yields a net separation of the experimental points at all concentrations, with the L‐Trp data  in the positive PC2 quadrants (with the exception of [L‐Trp] = 0 m), whereas all the D‐Trp data lie in the negative quadrants (apart from the point at [D‐Trp] = 1 pm, which nevertheless lies close to the zero line). Hence, a first criterium to account for the chirality sign at glance is to look at the resulting sign of the PC2. The identification of the enantiomeric form is unambiguous at concentrations higher than 10 pm: L‐Trp exhibits positive PC1 and PC2 scores, while D‐Trp exhibits positive PC1 and negative PC2 scores. The loading plot (green axes) displays the variables contributing to the PCs as vectors (green dashed arrows). The variables $S_{I_{DS}}$ and $S_{g_{m,l}}$ appear strongly correlated, within L‐Trp samples at concentration higher than 1 pm. In contrast, Δα is negatively correlated with D‐Trp samples, consistently with the observation that D‐Trp samples exhibit more negative Δα values. The parameter $-\frac{e\Delta V_{T}}{\sigma }$ is inversely correlated with [L‐Trp] > 1 pm suggesting a positive shift in the turn‐on voltage observed for L‐Trp binding. This is consistent with the increase in *I_DS_* current observed in the transfer characteristics upon exposure to Trp (as evident in Figure 3a).

To establish what is the minimum significant concentration (alike the LOD of a sensor) where the two enantiomers can be distinguished, we categorise the rescaled experimental data based on either the confidence or the prediction intervals. In Figure 4b the experimental score plot points are fitted with a parabola (dashed lines), and then we superimpose the prediction range estimated for 90% prediction level (shadowed bands). As a result, we are able to appreciate that the overlap of the prediction interval for L‐ a D‐ disappears at concentrations larger than 10 pm. If we take the same 90% level for the confidence interval, in alternative to prediction, the shadowed bands are separated already at 1 pm level. We infer that the LOD extracted with our approach for the enantiodiscrimination of L‐ and D‐Trp with EGOT is on the level of a few pm. These unprecedented results should be compared to the values reported earlier, where chiral discrimination levels were on the order of hundreds of µm when a chiral recognition group was assembled at the organic semiconductor channel and 200 pm when the gate electrode was functionalized with the odorant binding protein.

The outcome is that PCA alone can be effectively used to assign with low level of uncertainty the chirality of the enantiomer solution probed with the EGOT. We then want to show how the PCA jointly with the dose curves of the renormalized parameters can be effectively used to assign both the chirality and the concentration of blind samples. In Figure 4b we project the PC1 and PC2 scores obtained by combining the parameters ($S_{I_{DS}}\;$ at *V*_GS_ = −0.4 V, $S_{g_{m,l}}$, Δ*V*_T_, Δα) extracted from the transfer curves of two blind samples, on the PCA loadings reported in Figure 4a. The orange pentagon allows us to assign with no ambiguity the chirality to D‐Trp, as it falls into the range of prediction above 10 nm concentration. The blue star has an almost zero PC2 value and a strongly positive PC1 value which make the probability of the blind sample to belong to L‐Trp more likely. We can then extract the corresponding concentration from the dose curves. In the inset of Figure 4b we show where these points are placed in the dose curve of Δα as from Figure 3d. For the orange pentagon we find a concentration [D‐Trp] = 120 nm, and for the blue star a concentration [L‐Trp] = 130 nm, to be compared to nominal concentrations of the blind samples of 100 nm each.

The novel EGOT‐PCA method allows us to identify distinct clusters for each enantiomer. To assess the robustness of the approach, we applied PCA to the entire dataset comprising both L‐Trp and D‐Trp samples. We find that in this case the clustering on first two principal components is smeared and of no straightforward assignment. For overcoming this we look then to PC3 and PC4 for the entire data set. Figure S3a (Supporting Information) shows the PC3‐PC4 scores effectively separate the two chiral Trp forms. This demonstrates that the integration of multiple parameters can significantly enhance the analytical performance of the EGOT‐based biosensor even at the level of single measurements. The proposed classification model was validated using four independent experimental points within a concentration range from 1 pm to 1 nm. As shown in Figure S3b (Supporting Information), the two validation points for L‐Trp (blue stars) exhibit positive PC4 values, whereas the D‐Trp validation points (orange pentagon) are projected onto negative PC4 values.

To lower the level of discrimination of the two enantiomers we adopt a modified approach to PCA, where the whole calibration curves on the four characteristic variables in Figure 3, instead of the experimental data points, were input. In this manner we can exploit a much larger set obtained by combining 1000 points of each best fit dose curve vs concentration. This represents a predictive estimate of the PCs from the maximum likelihood data derived from the experimental data. The result of this novel PCA is shown in Figure 4c, where the score curves (continuous lines) are displayed: L‐Trp points (but the one at zero concentration) are all in positive PC2 quadrants, whereas the ones for D‐Trp are all negative. The prediction ranges at 95% confidence (shadowed bands) are extremely narrow, and they do not overlap as concentration reaches values as low as 100 fm. Hence, we infer that this “preprocessing” based on non‐linear regression of concentration dependent parameters from experimental data pushes further down the level of discrimination to sub‐pm level, *viz*., by one‐two orders of magnitude with respect to the standard PCA in Figure 4b. To increase the robustness of the enantiodiscrimination achieved through the innovative EGOT‐chemometric model, we applied a classification method – Partial Least Squares Discriminant Analysis (PLS‐DA) – to the entire dataset comprising both L‐Trp and D‐Trp samples. PLS‐DA extends the advantages of Partial Least Squares Regression (PLS‐R) to classification problems.^[^ ^75^ ^]^ Additional methodological details are provided in the Experimental Section. Specifically, we built a PLS regression model by correlating the experimental data extracted from the L‐Trp and D‐Trp transfer curves with a binary‐coded matrix representing class membership. This approach yielded, for each sample, a vector of predicted responses (Figure S4a, Supporting Information). The performance of the classification model was evaluated using Receiver Operating Characteristic (ROC) curves, shown in Figure S4b,c (Supporting Information) for L‐Trp and D‐Trp, respectively. In Figure S4d (Supporting Information), we propose an original visualization of the PLS‐DA model, in which the predicted response is plotted against the logarithm of the Tryptophan concentration, highlighting the response of the validation points (the same used for validation in the EGOT‐PCA classification, Figure S3b, Supporting Information).

In Figure 4d, we show the PCA score plot obtained from data of EGOT with EDC‐NHS functionalized gate. Again, the discrimination of enantiomers according to positive (L‐Trp) or negative (D‐Trp) values of PC1 in this case works with no ambiguity, however the correlation of the PCs with the concentration has largely faded out, leading to ambiguous results when PC1 is close to 0. This outcome, which seems surprising at first glance, reflects the insensitivity of the parameters Δα and Δ*V_T_* to Trp concentration in the case of the EDC‐NHS. This finding lets us infer that the multiparametric EGOT response is dependent on the functionalization strategy, and the more parameters are sensitive to the concentration, the more predictive the PCA can be.

## 3 Conclusion

We demonstrated an EGOT immunosensor for the detection of L‐Tryptophan across a wide concentration range from 1 fm to 10 nm. A quantitative analysis based on the multiparametric EGOT model of the transfer curves reveals that the Schiff Base Reaction (SBR) functionalization yields a narrower energy distribution of the recognition between antibody and target molecule. The evaluation of the dose curve based on a single parameter variation is not sufficient to discriminate the two enantiomers of Tryptophan. Hence, we devised a principal component analysis of the EGOT parameters enabling unambiguous clustering of the enantiomers at concentrations starting from 1–10 pm. These levels are four orders of magnitude smaller than the ones reported in label‐free discrimination of enantiomers and six orders of magnitude smaller than circular dichroism and magnetic dichroism. A further lowering of the level of enantio‐discrimination to sub‐pm concentration can be achieved with a modified PCA applied to best fit dose curves from the experimental parameters suitably renormalized. It is also shown that the level of discrimination and the predictivity of the method for concentrations of blind samples may depend critically on the functionalization strategy of the gate electrode.

As a perspective, the analysis we discussed can, in principle, be applied to assess the chiral purity of a mixture and/or enhance the analytical performance of EGOT biosensor when the target analyte is in real biological samples. We envision that the impact of such EGOT based platform, jointly with a suitable data analysis also powered by machine learning, can be useful in many points of use and benchtop practise, for instance for the high sensitivity analysis in drug synthesis, in pharmacology, pharmaco‐kinetics, in screening the presence of undesired enantiomers, or to sort out the occurrence of enzymatic reactions or metabolic products that may foster inversion of chirality.

## 4 Experimental Section

### Biomolecules and Chemicals

Purified monoclonal antibody for L‐Trp was purchased from Immusmol (Immunoglobulin type G) and resuspended in PBS1x pH 7.4 and stored at −20 °C.

6,13‐Bis(triisopropylsilylethynyl)pentacene, sulphuric acid (H_2_SO_4_, 96%), hydrogen peroxide (H_2_O_2_, 30%) aqueous glutaraldehyde (GA) solution (50%), sodium hydroxide, Ethanolamine, 6‐mercaptohexanol, 11‐mercaptoundecanoic acid, N‐(3‐dimemethylaminopropyl)‐N‐ethylcarbodiimide (EDC), N‐hydroxysuccinimide (NHS), ethanol, hexane, toluene, phosphate and chlorine salts were purchased from Merk‐Sigma‐Aldrich. 2‐Mercaptoehytamine hydrochloride (Cysteamine, CYS) were purchased from Thermoscientific.

### Device Fabrication

Source‐drain channel was fabricated as reported in ref. [47]. Briefly, test patterns were sonicated in ethanol for ten minutes, rinsed with water, and dried with a gentle flow of nitrogen (N_2_). A 1 µL droplet of a 1% (w/w) organic semiconductor solution in a 2:8 hexane: toluene mixture at 80 °C for 3 h, was drop‐cast onto the interdigitated electrodes and allowed to evaporate at room temperature.

### Gate Functionalization

To tailor the sensitivity of EGOT biosensor towards L‐Trp two different functionalization strategies have been investigated. For both techniques, the gate electrodes were sonicated 10 min in ethanol, rinsed with water, dried with a gentle flow N_2_, immersed in a hot (80 °C) piranha (H_2_SO_4_: H_2_O_2_ 1:1), rinsed abundantly with water, and dried with N_2_. For the SBR functionalization, the Au gate electrode was functionalised with cysteamine (30mm, in ethanol) and further treated with glutaraldehyde buffer (10%).^[^ ^60^, ^62^ ^]^ The functionalized electrode was incubated with L‐Trp Antibody solution (0.1 mg mL^−1^) at 4 °C. Ethanolamine solution (30 mm) was employed as passivating agent. For EDC‐NHS activated functionalization strategy the cleaned gate electrode was incubated overnight in a 10 mm mixture of Self‐Assembling Monolayer (SAM)‐forming molecules (11‐mercaptoundecanoic acid: 6‐mercaptohexanol 1:9 molar ratio) in ethanol at room temperature. The electrode was rinsed with ethanol, dried with N_2_ and incubated with 20 µL of 20 mm N‐(3‐dimemethylaminopropyl)‐N‐ethylcarbodiimide (EDC) and 50 mm N‐hydroxysuccinimide (NHS) (100 mm). After washing with water and drying with N_2_, the electrode was incubated with L‐Trp Antibody solution (0.1 mg mL^−1^) at room temperature and passivated with ethanolamine.

### Electrochemical Characterization

The gate electrode functionalization was characterized by CV and EIS performed in 5 mm K_3_[Fe(CN)_6_] and 1 m KCl. Both CV and EIS were collected using a Reference 620 potentiostat (Gamry Instruments, USA) and a three‐electrode cell: the gate electrode was used as working electrode, whereas a Pt wire and an Ag|AgCl (3 m KCl) were employed as counter electrode and reference electrode, respectively. For the EIS characterization, the electrode imaginary (Z″) and real (Z′) electrochemical impedance responses were recorded at an AC voltage amplitude of 10 mV and DC potential of 0.26V, in a frequency range from 0.1 Hz and 10 kHz. For CV, a scan rate equal to 100 mV s^−1^ was chosen.

### Electrical Characterization

For electrical measurements, source, drain, and gate electrodes were connected to an Agilent B2902A Source Measure Unit. Gate electrodes were incubated in situ for 15 min, in solutions containing increasing concentration of the analyte. The device was characterized by sweeping the gate‐source voltage (V_GS_) from −0.1 to −0.7 V, while the drain‐source voltage (V_DS_) was kept constant at −0.2 V. After each step of functionalization and sensing, the devices were measured until stabilization. To compare data, the following procedure was adopted for the stabilization. Gate bias sweep and channel current continuous recording were applied for at least 60 min to extract the transfer curves. A device is considered stabilized when three consecutive transfer curves are superimposed else they overlap within a standard deviation smaller than 1 nA at V_GS_ = −0.7 V.

### Enzyme‐Linked Immunosorbent Assay (ELISA)

The commercially available ELISA kit for L‐Tryptophan (BA‐E‐2700R‐F ELISA) was purchased from Immusmol. The ELISA assay was carried out in PBS solution, testing Trp concentration within 10 µm to 0.15 mm concentration range and using a Perkin Elmer Victor X as multilabel plate reader platform.

### Atomic Force Microscopy

The surface morphology of the functionalized gate electrodes was investigated using a Park XE7 AFM System (Park Systems, Suwon, Korea), operating in tapping mode, in air, at room temperature. The instrumentation features pre‐mounted silicon cantilevers (OMCL‐AC160TS, Olympus Micro Cantilevers, Tokyo, Japan) with reflective coatings, tip curvature radius of ≈7 nm, elastic constant of ≈26 N m^−1^, and resonance frequency of ≈300 Hz. The topographic images (1,024 pixels, dimensions: 5 µm × 5 µm) were analyzed using Park Systems XEI software (Park Systems, Suwon, Korea) and Gwyddion freeware (version 2.62 http://gwyddion.net/).

### Chemometric Data Analysis

Principal component analysis (PCA) was used in bioanalytical science to reduce a large amount of data into few parameters. known as the principal components (PCs), capturing the differences and similarities among the samples and the variables of the original dataset.^[^ ^76^ ^]^ Briefly, PCs, calculated by solving an eigenvalue/eigenvector problem, are linear combinations of the variables in the original dataset and therefore are orthogonal each other. PCA decomposes the data matrix by coupling score vectors, giving the coordinates of the samples (variables) in the PCs space, in order to maximize the distances between points. Loading vectors represent the weight assigned to each original variable contributing to the PCs.^[^ ^77^ ^]^ Here, the turn‐on voltage shift Δ*V_T_*, rescaled vs σ, the α parameter shift (*Δα*) and the signals $S_{g_{m,l}}$, and $S_{I_{DS}}$ at *V*_GS_ = −0.4 V were used as basis set for PCs calculation. PCA calculation was performed using singular value decomposition algorithm processed by PLS Toolbox 8.9.1 (2024) (Eigenvector Research, Inc., Manson, WA USA 98831 software). Prior to the development of PCA decomposition, the input basis set underwent autoscaling (*viz*., adjusted to zero mean and unit variance by dividing each column by its standard deviation). Leave‐one‐out cross‐validation method was used to assess the optimal number of principal components. Partial least square discriminant analysis (PLS‐DA) is a supervised, multivariate, dimensionality‐reduction method for classification.^[^ ^75^, ^78^ ^]^ In the input arrays for PLS‐DA, the parameters ($\frac{e\Delta V_{T}}{\sigma }$, *Δα*, $S_{g_{m,l}}$, and $S_{I_{DS}}$ at *V*_GS_ = −0.4 V) extracted from transfer curves of all the L‐Trp and D‐Trp samples were used as set of predictors. These samples were used as training set with a binary‐coded column vector that encoded the classification information (0 for L‐Trp and 1 for D‐Trp). The classification model was assessed with Receiver Operating Characteristics Curve (ROC curve), which reports the specificity (number of samples predicted as not in the class divided by actual number not in the class) versus the sensitivity (number of samples predicted as in the class divided by number actually in the class). The model was further evaluated with four independent experimental points within a concentration range of 1 pm to 1 nm.

## Conflict of Interest

The authors declare no conflict of interest.

## Author Contributions

M.G. performed the investigation, data curation, methodology, wrote the original draft, wrote the review, and performed editing. P.G. performed conceptualization, data curation, formal analysis, supervision, wrote the original draft, wrote the review, and performed editing. A.P. performed the investigation, methodology, wrote the review, and performed editing. M.S. performed the investigation, methodology, wrote the review, and performed editing. M.B. performed the investigation, methodology, wrote the review, and performed editing. M.D.L. performed data Curation, methodology, and wrote the original draft. M.M. performed data curation, methodology, supervision, and wrote the original draft. C.A.B. performed data curation, methodology, supervision, and wrote the original draft. L.F. performed methodology, validation, supervision, funding acquisition, wrote the review, and performed editing. F.B. performed conceptualization, formal analysis, validation, supervision, funding acquisition, wrote the original draft, wrote the review, and performed editing.

## Supporting information



Supporting Information

## Data Availability

The data that support the findings of this study are available from the corresponding author upon reasonable request.
