# Supplementary material for: Discrimination of Tryptophan Enantiomers at Sub‐pm Level by Multiparametric Analysis of a Label‐Free Organic Immunosensor
Source: Small Methods. 2025 Aug 15;10(3):2500545. doi: 10.1002/smtd.202500545 (PMC12893247; doi:10.1002/smtd.202500545)
Supplement: Supplementary file 1 — Supporting Information [file SMTD-10-2500545-s001.docx]

**Discrimination of Tryptophan Enantiomers at sub-pM level by**

**Multiparametric Analysis of a Label-free Organic Immunosensor**

Matteo Genitoni^a,b^, Pierpaolo Greco^a,b,d*^, Alessandro Paradisi^a,c^, Matteo Sensi^c,d^, Marcello Berto^a,c^, Mauro Murgia^a,d,e^, Michele Di Lauro^a,d^, Carlo Augusto Bortolotti^c^, Luciano Fadiga^a,b,d^, Fabio Biscarini^a,c,d*^

*^a^ Center for Translational Neurophysiology of Speech and Communication, Istituto Italiano di Tecnologia, Ferrara, 44121, Italy.*

*^b^ Department of Neuroscience and Rehabilitation, Università di Ferrara, Ferrara 44121, Italy.*

*^c^ Life Science Department, Università di Modena e Reggio Emilia, Modena, 41125, Italy.*

*^d^ Organic Bioelectronics Srl, Ferrara, 44121, Italy.*

*^e^ Institute for the Study of Nanostructured Materials (ISMN), Consiglio Nazionale delle Ricerche, Bologna, 40129 Italy.*

| ***Techniques*** | ***Stereo-selective material*** | ***Transducer material*** | ***[Trp] range*** | ***Ref*** |
| --- | --- | --- | --- | --- |
| ***Chromatography :***  High-Performance Liquid Chromatography | o-Phthalaldehyde, N-acetyl-L-cysteine | Chemiluminescence | 1.2 μM – 59 μM | ^[15]^ |
|  | Teicoplanin chiral stationary phase | Diode array detector | 24.5 μM – 2.5 mM | ^[16]^ |
|  | (−)-(18-crown-6)-2  3, 11, 12-tetracarboxylic acid (Chirosil-SCA) | Photodiode array detector | 450 μM – 3.3 mM | ^[17]^ |
| ***Capillary Electrophoresis (CE):***  Capillary Electrophoresis | n.g. | Circular dichroism | n.g. | ^[19]^ |
| ***Mechanical:*** |  |  |  |  |
| Microcantilevers deflection | Monoclonal anti-Trp antibody | Nanostructured microcantilever | 2.5 μM | ^[66]^ |
| Temperature | N-Acetyl-L-Cysteine | Mxene-Au NPs | 1.0 mM - 6.0 mM | ^[67]^ |
| ***Optical:*** |  |  |  |  |
| SERS-spectroscopy | 6-mercapto-6-deoxy-β-CD | TiO_2_ NPs | 100 nM - 1 mM | ^[23]^ |
| UV-spectroscopy | ZnFe2O4-L-Cys | n.g. | 0.020 pM - 0.090 μM | ^[25]^ |
| Surface Plasmon Resonance | Polyclonal antibody-anchored to enantiomeric pure ligand | n.g. | 3 nM- 500 mM | ^[24]^ |
| Colorimetric sensor | Zif-8 His MOF MIP, L-Tartaric acid-capped Au NPs | Nano-ovals shaped Au NPs, n.g. | 250 μM - 1.25 mM | ^[18,68]^ |
| Fluorescence | Self-Assembled fluorescence nanoprobe,  CD and Bovine Serum Albumin | n.g. | 1 μM - 6 mM | ^[69,70]^ |
| ***Electrochemical:*** |  |  |  |  |
| Cyclic voltammetry (CV) | Poly-L-Cys MWCNTs; L-alanine ethyl ester modified carbon MWCNTs | GCE | 0.1 mM - 5.0 Mm | ^[71,72]^ |
| Electrochemical impedance spectroscopy (EIS) | Au-Ag nanoparticles | GCE | 0.1 mM – 10 mM | ^[73]^ |
| Differential pulse voltammetry (DPV) | L-Lysine based MOF | Ferrocene-modified  MWCNTs | 2 μM - 100 μM | ^[74]^ |
|  | N-acetyl-L-cysteine | MXene-Au NPs | 0.2 mM - 1.0 mM | ^[67]^ |
|  | MWCNTs self-assembled with Cu^2+^-β-CD; β-CD modified with PL-Cys | GCE | 3 μM - 4 mM | ^[71,73]^ |
|  | Sodium alginate - Chitosan | N-doped graphene CNT-GCE | 5 mM | ^[75]^ |
|  | Sodium carboxymethyl cellulose; Copper-Cys mercaptide nanorods; Glutamic acid functionalized graphene-gold NPs, MIP/CS/MWCNTs/ | GCE | 1 μM - 7 mM | ^[20–22,76]^ |
| CV-DPV | Biomimetic MOF, Polyaniline twisted nanoribbon | GCE | 0.2 mM - 5 mM | ^[77,78]^ |
| Transistor | MIP-modified gate electrode | PEDOT:PSS-based EGOT | 1 nM - 3 μM | ^[27]^ |
|  | Nafion GO functionalized with BSA | MOSFET | 1 pM - 1μM | ^[26]^ |
|  |  |  |  |  |
|  |  |  |  |  |

**Table 1SI:** summary of the Tryptophan sensors exploiting different transduction techniques and material for enantiorecognition of Tryptophan.


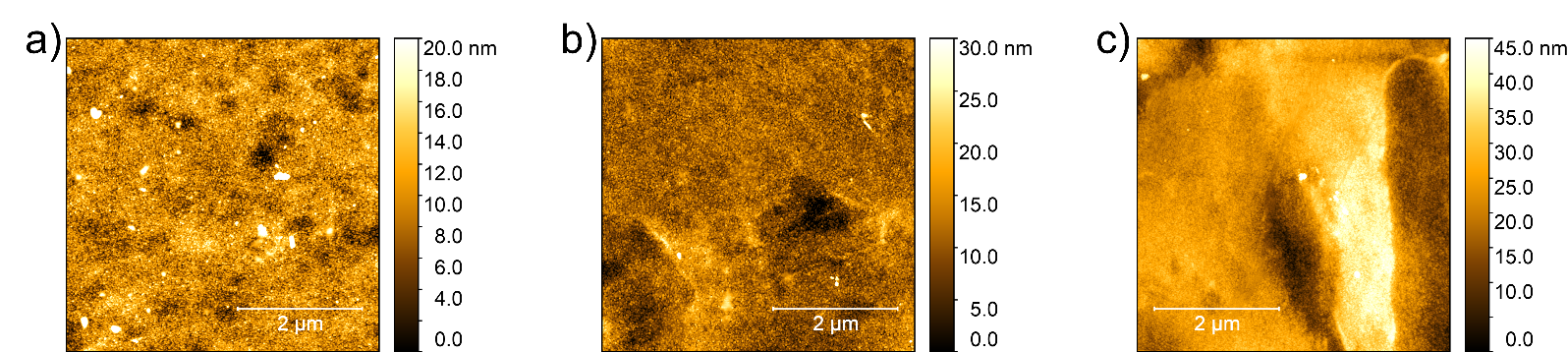
***Figure 1SI****: AFM images of* ***a)*** *bare gold electrode after cleaning procedure;* ***b)*** *morphology of EDC-NHS-functionalized electrode;* ***c)*** *morphology of electrode activated with SBR functionalization showing wavy feature.*

**Table 2SI:** parameters extracted by fitting the dose response of different parameters ($S_{I_{DS}}$, $S_{g_{m,l}}$, ${\Delta V}_{T}$, $\Delta\alpha$) with Universal Langmuir Model (ULM).


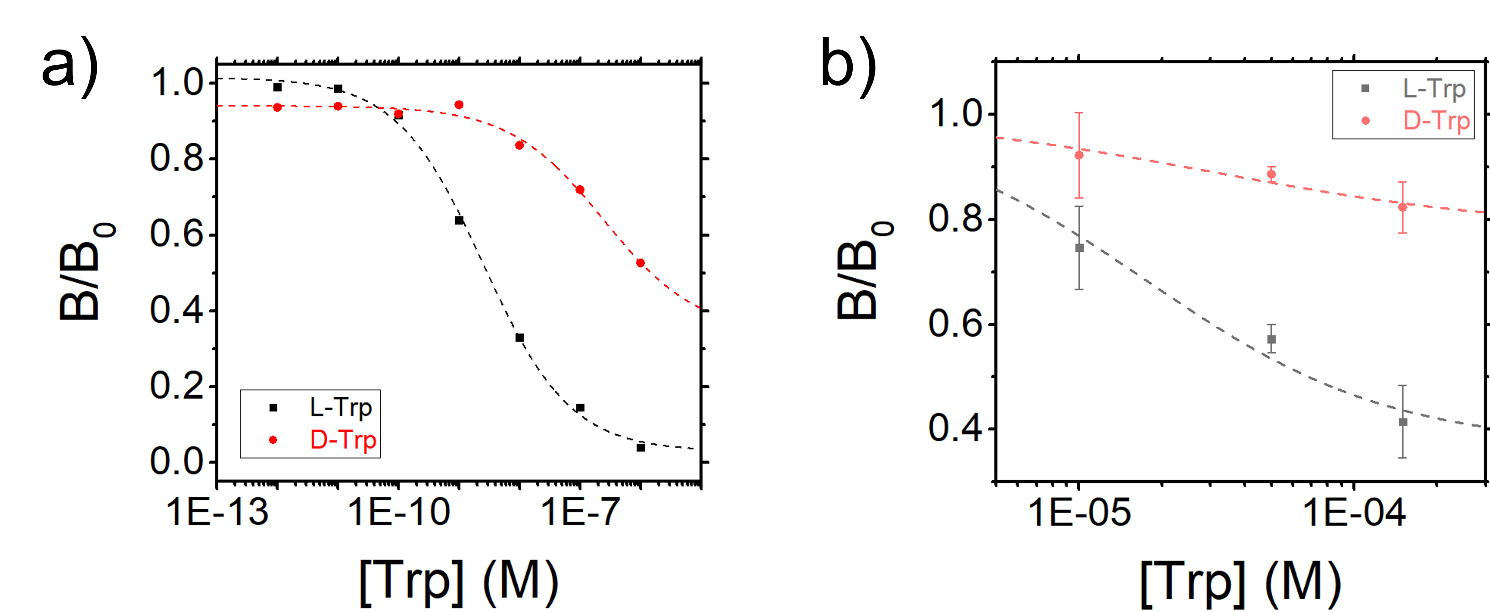


***Figure 2SI****:* ***a)*** *Normalized results obtained from the ELISA test for L-Trp (black squares) and D-Trp (red circles), performed using the mouse monoclonal anti-L-Trp antibody (the same used in the EGOT immunosensor). The sensitivity range is between 1 pM and 1 μM. Dashed lines represent the best-fit curves obtained using the following expression:* $\frac{B}{B_{0}}=START+(END-START)(1-\frac{{K_{A}}^{n}\left[ Trp \right]^{n}}{1+{K_{A}}^{n}\left[ Trp \right]^{n}})$ ***b)*** *Normalized results obtained from the ELISA test using the rabbit polyclonal anti-L-Trp antibody for L-Trp (grey squares) and D-Trp (light red circles), within the sensitivity range declared by the manufacturer (from 10 μM to 1 mM).*


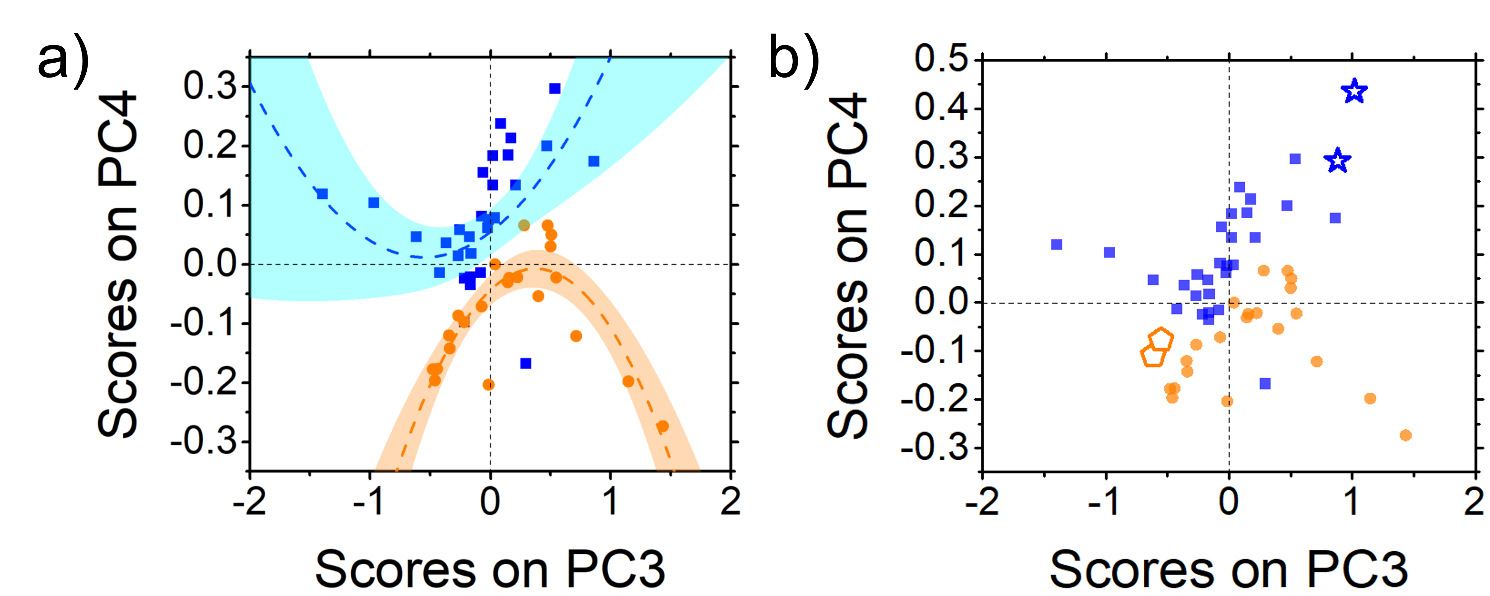
***Figure 3SI****:* ***a)*** *The score plot of PC3 versus PC4 obtained from PCA analysis carried out on the entire dataset for L-Trp (blue squares) and D-Trp (orange circles). The clusterization of the two enantiomeric forms is highlighted by the parabolic fit (reported with 95% confidence bands, shadowed areas) included as a guide-to-the-eye;* ***b)*** *The projection of the PC3 vs PC4 scores of four independent datasets (blue stars for L-Trp, orange pentagon for D-Trp) for the PCA-EGOT model validation.*


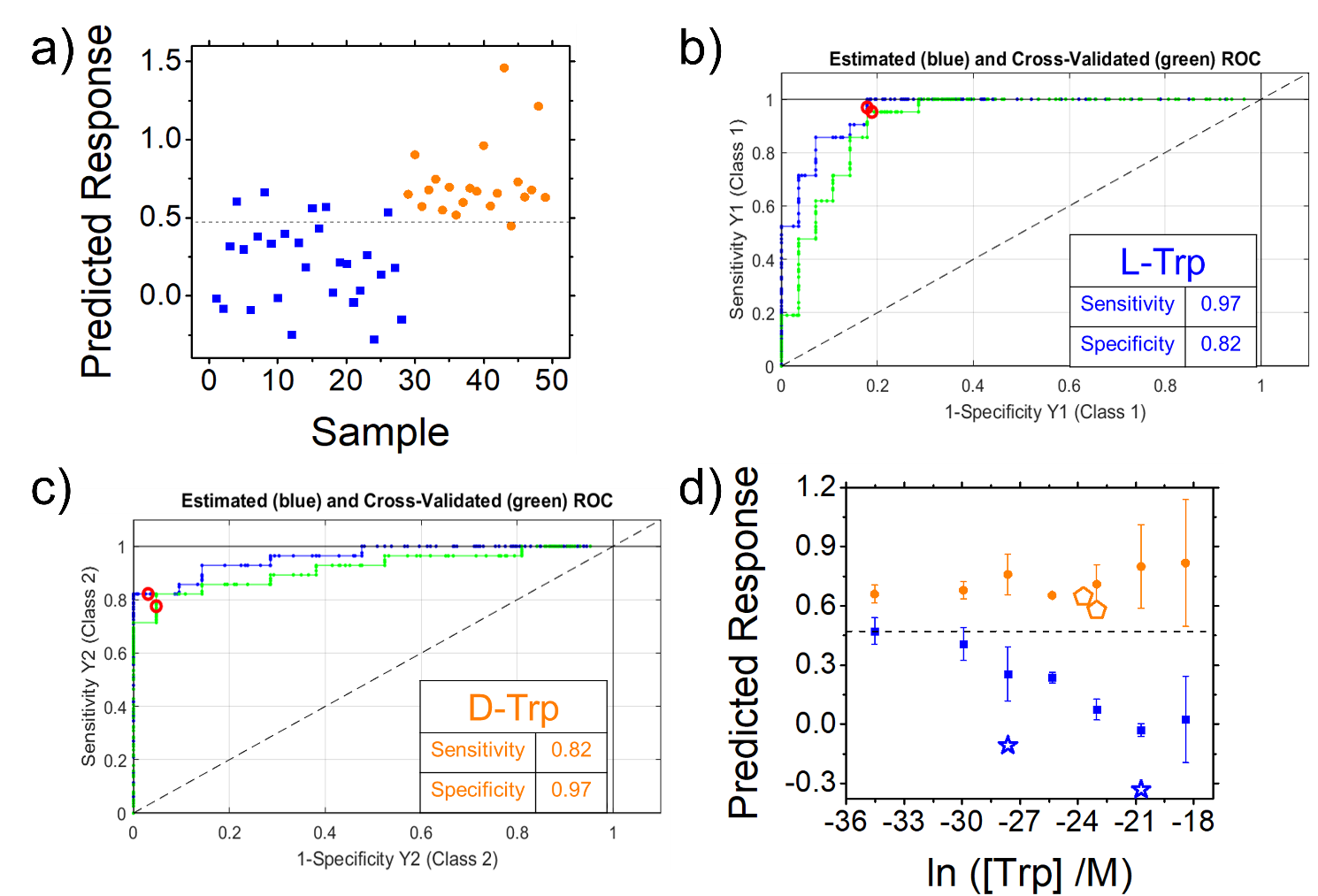


***Figure 4SI****:* ***a)*** *Illustration of PLS-DA classification model based on predicted response calculated for L-Trp (blue squares) and D-Trp (orange circles) at all concentrations. The black dashed line represents the calculated classification threshold (y=0.47): samples falling above the line are assigned to D-Trp, while samples falling below are assigned to L-Trp.* ***b-c)*** *Receiver Operating Characteristic (ROC) curves for PLS-DA model for L-Trp (****b****) and D-Trp (****c****) samples.* ***d)*** *Plot of predicted response against the concentration to highlight the correct association of four independent validation points to enantiomeric group (blue stars for L-Trp and orange pentagons for D-Trp).*
